# Syphilis screening and treatment: integration with HIV services

**DOI:** 10.2471/BLT.17.200923

**Published:** 2017-09-01

**Authors:** Melanie M Taylor, Mary Kamb, Dadong Wu, Sarah Hawkes

**Affiliations:** a Department of Reproductive Health, World Health Organization, avenue Appia 20, 1211 Geneva 27, Switzerland.; b Division of Sexually Transmitted Diseases Prevention, Centers for Disease Control and Prevention, Atlanta, United States of America.; c Institute for Global Health, University College London, London, England.

Syphilis transmitted from mother to child is second only to malaria as a leading cause of preventable stillbirth.^1^ An estimated 930 000 pregnant women experience 350 000 adverse pregnancy outcomes annually due to syphilis. Over half of these affected pregnancies end in stillbirth or neonatal death.^2^ Elimination of mother-to-child transmission of syphilis relies on reducing the background prevalence of syphilis in pregnant women as well as within general populations. Screening and treatment of high-risk populations and pregnant women are critical strategies that can rapidly reduce morbidity and mortality related to this infection. However, while countries frequently have well-resourced programmes for human immunodeficiency virus (HIV) prevention and care, syphilis control is seldom centralized and often falls within the responsibilities, but outside the priorities, of HIV programmes or antenatal care. Advocacy, policy implementation and resource allocation for syphilis testing and treatment are often deficient, particularly in countries with high syphilis burden.^3^

The World Health Organization (WHO) strategy for the elimination of congenital syphilis prioritized commitment and advocacy at the highest levels, alongside promoting increased access to and quality of maternal and newborn health services, improved coverage of syphilis testing and treatment in pregnancy and ongoing surveillance, monitoring and evaluation of targets.^4^ In 2014, regional and country-led initiatives as well as encouragement by external partners led WHO to promote integrating programmes for dual elimination of mother-to-child transmission of both HIV and syphilis, recognizing that harmonized approaches improved efficiency and quality of maternal and newborn health services and improved health outcomes for mothers and infants. The resulting WHO global guidance on elimination of mother-to-child transmission of HIV and syphilis set corresponding process criteria for country validation of (i) at least 95% coverage of antenatal care; (ii) 95% testing coverage of pregnant women for HIV and syphilis; and (iii) 95% treatment coverage for those pregnant women testing positive for HIV or syphilis.^5^ In addition to antenatal-care-specific targets, the global guidance also calls for enhanced access for the general population to syphilis prevention and control interventions.

Integration of syphilis prevention with HIV control programmes offers the opportunity to expand coverage of syphilis testing and treatment among populations at risk for both infections, particularly among pregnant women, who bear the highest burden of adverse outcomes. Aligning elimination efforts may also contribute to strengthening the health system and saving costs.^6^ For example, in China, syphilis was low on the national policy agenda due to limited external support, uncoordinated domestic initiatives, lack of awareness of the problem and its solutions among policymakers and negative issue framing leading to stigma and discrimination.^7^ Since 2010, in China, syphilis screening and treatment has been gradually incorporated into HIV counselling and testing sites,^8^ and integrated antenatal HIV and syphilis interventions have become a normal part of routine antenatal care.^9^ Leveraging the well-established platform of HIV control, including health facilities, staff, supplies and data reporting mechanisms has proven effective not only in enhancing political commitment to combat syphilis but also in mobilizing resources for the expansion of syphilis testing and treatment. According to data from the National Center for Women and Children’s Health in China, the annual number of pregnant women tested for syphilis increased rapidly, and by 2013 over 96% of antenatal care attendees in areas covered by the integrated programme were tested for syphilis and HIV.^9^

New technology has increased the feasibility of integrated testing. For example, the development of rapid dual HIV/syphilis test kits has enabled additional syphilis intervention opportunities within clinical settings. This kit allows simultaneous testing for both infections on a single cartridge. A systematic review has shown that the field performance of rapid dual HIV/syphilis tests is comparative to laboratory reference testing.^10^ The added utility and feasibility of a single platform for testing of multiple infections makes these tests attractive for use among populations recommended for routine screening for both infections. WHO recognizes dual HIV/syphilis (multiplex) point-of-care diagnostics as an option for HIV testing and has released interim guidance on using and interpreting these tests, pending a WHO-approved algorithm.^11^ In some areas, HIV resources may be leveraged to purchase rapid dual HIV/syphilis test kits.

While improved diagnostic tools may improve efficiency and testing coverage, securing and maintaining strong political commitment is also critical to programme success. China has succeeded in increasing syphilis testing and treatment for pregnant women nationally, and for high-risk groups in some areas, by strategically integrating syphilis control with HIV – an issue that receives both political attention and resources in the country.^8^^,^^9^^,^^12^ Such integration may be reproducible in other countries, particularly those where syphilis burden is high. However, integration cannot rely on technology alone. As the experience of China has shown, elimination of mother-to-child transmission of both HIV and syphilis relies upon the commitment of policy-makers and associated resource allocation, both of which are likely to be driven by sustained advocacy to promote comprehensive and effective antenatal care.
